# The Phylosymbiosis Pattern Between the Fig Wasps of the Same Genus and Their Associated Microbiota

**DOI:** 10.3389/fmicb.2021.800190

**Published:** 2022-02-14

**Authors:** Jiaxing Li, Xianqin Wei, Dawei Huang, Jinhua Xiao

**Affiliations:** College of Life Sciences, Nankai University, Tianjin, China

**Keywords:** insect, host-associated microbiota, holobiont, 16S rDNA, coevolution, *Wolbachia*

## Abstract

Microbial communities can be critical for many metazoans, which can lead to the observation of phylosymbiosis with phylogenetically related species sharing similar microbial communities. Most of the previous studies on phylosymbiosis were conducted across the host families or genera. However, it is unclear whether the phylosymbiosis signal is still prevalent at lower taxonomic levels. In this study, 54 individuals from six species of the fig wasp genus *Ceratosolen* (Hymenoptera: Agaonidae) collected from nine natural populations and their associated microbiota were investigated. The fig wasp species were morphologically identified and further determined by mitochondrial CO1 gene fragments and nuclear ITS2 sequences, and the V4 region of 16S rRNA gene was sequenced to analyze the bacterial communities. The results suggest a significant positive correlation between host genetic characteristics and microbial diversity characteristics, indicating the phylosymbiosis signal between the phylogeny of insect hosts and the associated microbiota in the lower classification level within a genus. Moreover, we found that the endosymbiotic *Wolbachia* carried by fig wasps led to a decrease in bacterial diversity of host-associated microbial communities. This study contributes to our understanding of the role of host phylogeny, as well as the role of endosymbionts in shaping the host-associated microbial community.

## Introduction

Microbes play important roles in hosts’ biology. All insects are colonized by microbes, and the microbiota accounts for 1–10% of the insect’s biomass (Douglas, 2015). Bacterial symbionts have been accepted by biologists as considerable drivers of insect nutrition, protection, detoxification, behavior, reproduction, communication, and evolution (Douglas, 2015; Salcedo-Porras et al., 2020). Historically, research on symbiotic relationship between insects and microorganisms has focused mainly on insect hosts and their obligate bacterial symbionts, such as aphids and *Buchnera aphidicola* (Chong et al., 2019), whiteflies and *Portiera aleyrodidarum* (Santos-Garcia et al., 2020), psyllids and *Carsonella ruddii* (Thao et al., 2000), and mealybugs and *Tremblaya princeps* (Lopez-Madrigal et al., 2015), or focused on specific endosymbionts known to be widespread in arthropods, such as *Wolbachia* (Hertig and Wolbach, 1924) or *Spiroplasma* (Davis et al., 1972; Cisak et al., 2015). However, symbioses are formed by complex multi-part interactions, including interactions between the hosts and their resident microbiota as well as interactions within the microbial community. Under these circumstances, binary interactions between hosts and endosymbionts were broadened to multivariate interactions between hosts and all microbes of their associated microbial community, as they are so important to the hosts (Brinker et al., 2019). A host organism and its associated microbial community form an entity that is termed as holobiont, which is considered as a unit of selection in evolution (Rosenberg et al., 2007). This understanding clarifies the holobiont as a complex ecosystem in which the host and associated microbiota are closely linked.

A deluge of data has enabled unprecedented insights into the extensive taxonomic, genetic, and functional compositions of host-associated microbial communities (Lim and Bordenstein, 2020). However, our understanding of the interaction between microbiota and host is still relatively superficial due to quantitative limiting factors, such as incredibly diverse interactions between microbiota and host, and the technical limitations that plenty of microbes *in vivo* are difficult to be isolated and cultured *in vitro* (Lagier et al., 2018). It is generally considered that the host-associated microbiota can be shaped primarily by diets, environment, and host phylogeny (Kartzinel et al., 2019). In addition, interactions within microbiota can also shape the diversity and structure of insect bacterial communities (Guegan et al., 2018), in which the endosymbionts *Wolbachia* and *Spiroplasma* are widely studied. For example, parts of the native mosquito microbiota can impede vertical transmission of *Wolbachia* in *Anopheles* (Hughes et al., 2014). In *Drosophila melanogaster*, *Spiroplasma* reduced *Wolbachia* density but not vice versa (Goto et al., 2006), while in spider mite, *Wolbachia* dominated relative to *Spiroplasma* (Yang et al., 2020). These observations suggest exclusion or competition within microbiota. Unfortunately, data on microbial interactions are still scarce, and the mechanisms involved remain unclear.

In recent years, a new term phylosymbiosis has been proposed to describe the interaction between host and associated microbiota (Brucker and Bordenstein, 2013). According to the study of phylosymbiosis, host-associated microbial community relationships recapitulate the phylogeny of their hosts, indicating that the relationships of microbiota across host species maintain an ancestral signal of the host’s evolution (Brucker and Bordenstein, 2013). Therefore, host phylogeny can reflect or be reflected by microbial community structure. In other words, phylosymbiosis may reveal whether there is an interaction between host phylogeny and bacterial community. Coevolution, cospeciation, codiversification, or cocladogenesis may lead to phylosymbiosis, and this pattern may alternatively arise by antagonistic interactions and/or horizontal transmission of the microbiota due to the direct microbial transfer between related individuals (Lim and Bordenstein, 2020). To date, a great deal of phylosymbiosis systems have been explored, such as mammals (Groussin et al., 2017), omnivorous cockroaches (Tinker and Ottesen, 2020), widow spider (Dunaj et al., 2020), freshwater snails (Huot et al., 2019), coral reef fish (Chiarello et al., 2018), and their associated microbiota. However, the host taxa in these studies were mainly across orders, families, or genera. Due to the long-term differentiation and distant genetic relationship between hosts, their microbial communities are distinct. Therefore, the phylosymbiosis patterns are easily detected in the case of the studies described above. In the lower taxa, within a genus for instance, different species are phylogenetically closely related, and according to the studies of phylosymbiosis, the more closely related the taxa are, the more similar the compositions of their associated microbial communities are (Brucker and Bordenstein, 2012a,b; O’Brien et al., 2019). In a previous study, phylosymbiosis signals were observed in salamanders and frogs at the taxonomic levels of order, but were not observed within genera and species (Ellison et al., 2019). There are few studies on phylosymbiosis within genera, and even if there are, the subjects were mostly lab-fed organisms, such as *Nasonia* wasps (Brucker and Bordenstein, 2012a,^2013^), *Drosophila* flies, and *Peromyscus* deer mice (Brooks et al., 2016).

In this study, we collected fig pollinators (Hymenoptera: Agaonidae) to explore their microbiota and test whether a phylosymbiosis signal between insect hosts and their associated microbiota is prevalent among closely related species within a genus. Fig-pollinating wasps are the only pollinators of fig trees (*Ficus*, Moraceae) (Corner, 1958; Berg, 1989), and they spend almost their whole lives in fig fruits (syconia), which is a relatively closed and stable system. The larvae of fig pollinators only feed on the galled fig flowers (Janzen, 1979), so their diets are simple. The characteristic life histories and diets make the fig pollinators and their microbial community an excellent model to experimentally investigate evolutionary dynamics of host-microbiota interactions. In addition, endosymbiont *Wolbachia* is highly prevalent in fig pollinators (Chen et al., 2010), which allows us to investigate the effects of the endosymbiont *Wolbachia*, the phylogenetic relationship of fig wasp hosts, and the unique symbiotic environment provided by fig fruit on the host bacterial communities.

In this study, six fig pollinator species of the genus *Ceratosolen* and their microbial community were investigated. The mitochondrial cytochrome c oxidase subunit 1 (CO1) gene fragment and nuclear marker internal transcribed spacer 2 (ITS2) sequences were used to reconstruct the phylogenetic relationship of nine populations of the six species. The 16S ribosomal DNA amplicon sequencing was used to analyze the bacterial communities of these nine populations. We detected significant phylosymbiosis signal between the fig pollinators and their microbial communities. Our results also showed that *Wolbachia* was the dominant bacteria in the infection samples, and the fig wasps infected with *Wolbachia* had a lower bacterial diversity than those not infected. These results revealed the phylosymbiosis relationship between hosts and microbial communities in natural insect populations at a low taxon level, and the effect of the endosymbiotic *Wolbachia* on shaping host microbial communities.

## Materials and Methods

### Sample Collection

Figs of *Ficus semicordata*, *Ficus racemosa*, *Ficus tikoua*, *Ficus fistulosa, Ficus auriculata*, and *Ficus hispida*, which are hosts of *Ceratosolen gravelyi*, *Ceratosolen fusciceps*, *Ceratosolen* sp., *Ceratosolen hewitti*, *Ceratosolen emarginatus*, and *Ceratosolen solmsi*, respectively, were collected from Yunnan, Hainan, Guangxi, Guizhou, and Guangdong provinces in China (Supplementary Table 1). The figs we collected were wiped with alcohol cotton balls before being placed in sterile plastic cups. Each fig was put in a plastic cup and reserved in a climate chamber (humidity 70%, 16:8 h/L: D, 25°C/25°C). We collected adult fig wasps as soon as we found them emerging from figs, and then collected every 5–10 min for about 5 h per fig. The wasps collected were put in ethanol and stored at −20°C refrigerator. We collected dozens or even hundreds of fig pollinators per fig, and then randomly selected the unwounded female individuals that do not lack appendages for the experiment. The fig wasps immersed in ethanol were identified and selected under stereozoom microscope (Motic SMZ-168), and the identification was confirmed by CO1 gene fragment and ITS2 sequences (as described below). Fig wasps of nine populations from six species were used in the following analysis, with six samples from each population.

### DNA Extraction and PCR Amplification

Total genomic DNA from each fig wasp was extracted with DNeasy Blood and Tissue Kit (Qiagen, Germany) according to the protocol. Single fig pollinator was washed three times with sterile water before the extraction of genomic DNA. DNA concentration and purity were monitored on 1% agarose gels. According to the concentration, DNA was diluted to 1 ng/μl using sterile water and stored at −20°C refrigerator. The host CO1 gene was amplified by primers LCO1490 (5′-GGTCA ACAAATCATAAAGATATTGG-3′) and HCO2198 (5′-TAAA CTTCAGGGTGACCAAAAAATCA-3′) (Folmer et al., 1994). The nuclear marker ITS2 was amplified by four pairs of primers. Specifically, the ITS2 of *C. gravelyi*, *C. emarginatus*, and *Ceratosolen* sp. was amplified by primers 5.8s-Fc (5′-TGAACATCGACATTTYGAACGCACAT-3′) and 28S-D4-5R (5′-GTTACACACTCCTTAGCGGA-3′) (Cruaud et al., 2010). The ITS2 of *C. solmsi*_2 was amplified by primers Aed5.8F (5′-GTGAACTGCAGGACACATGAAC-3′) and AedAB28 (5′-ATATGCTTAAATTTAGGGGGT-3′) (Kjer et al., 1994; Brust et al., 1998). The ITS2 of *C. solmsi*_1 and *C. fusciceps* was amplified by primers designed based on *C. solmsi* genome (accession number: PRJNA277475), namely, ITS2-L11F (5′-TTTGCGCGTCAACTTGTGAA-3′) and ITS2-L11R (5′-TCG CCGCTACTGAGGAAATC-3′), and ITS2-L9F (5′-GCAGG ACACATGAACATCGAC-3′) and ITS2-L9R (5′-TCTCAA GCAACCCGACTCTG-3′), respectively. The ITS2 sequence of *C. hewitti* failed to be amplified using the primers described above. PCR reaction system included 5 ng DNA template, 2.0 μM each primer, 0.2 mM dNTPs, 1.25 U of *EasyTaq*^®^ DNA Polymerase, and 1 × *EasyTaq*^®^ Buffer (TransGen Biotech, Beijing, China), then sterile water was added to a total volume of 25 μl. The PCR conditions of CO1 consisted of 5 min at 94°C, 35 cycles of 94°C for 30 s, 51°C for 45 s, 72°C for 1 min, and 10 min at 72°C. The amplification of ITS2 followed the protocol outlined for CO1 above, but 40 cycles of amplification, an annealing temperature of 54°C, and an extension time of 2 min and 30 s were employed. Blank controls were set during DNA extraction and PCR amplification to exclude contamination. Finally, the PCR products were purified and sequenced by conventional Sanger sequencing (Sangon Biotech, Shanghai, China).

### Detection of *Wolbachia* in Fig Wasps

The presence/absence of *Wolbachia* in a fig wasp was detected by a PCR-based assay with *Wolbachia*-specific primers. Three pairs of *Wolbachia*-specific primers including wsp81F (5′-TGGTCCAATAAGTGATGAAGAAAC-3′) and wsp691R (5′-AAAAATTAAACGCTACTCCA-3′) (Zhou et al., 1998), FtsZ-F (5′-TACTGACTGTTGGAGTTGTAACTAACGCGT-3′) and FtsZ-R (5′-TGCCAGTTGCAAGAACAGAAACTCTAACTC-3′) (Jeyaprakash and Hoy, 2000), and 16SwolF (5′-TTGTAGC CTGCTATGGTATAACT-3′) and 16SwolR (5′-GAATAGGTA TGATTTTCATGT-3′) (O’Neill et al., 1992) were used. Each fig wasp was individually diagnosed with *Wolbachia*. Amplifying conditions were the same as used in the CO1 gene except for the annealing temperature, which were 55, 55, and 47°C for *wsp*, *fts*Z, and 16S rRNA gene, respectively. Blank control was set during PCR amplification, and sterile water of equal volume was used instead of template. Amplified fragments were revealed under UV light after migration on 1% agarose gel electrophoresis. A fig wasp was confirmed to be infected with *Wolbachia* only when all three genes were successfully amplified. Similarly, if none of the three genes could be amplified, the individual was considered not infected with *Wolbachia*.

### Phylogenetic Reconstruction of Pollinators and Fig Trees

Phylogenetic reconstruction of insects was performed by combining CO1 fragments and ITS2 sequences. We obtained CO1 sequences for all 54 samples and 40 ITS2 sequences (at least one for each population except *C. hewitti*). The genetic distance over CO1 and ITS2 sequences between populations was estimated with MEGA7 (Tamura and Nei, 1993; Kumar et al., 2016). For phylogenetic trees, a fig pollinator species of *Kradibia gibbosae* was set as the outgroup and its mitochondrial sequence containing CO1 was downloaded from GenBank (accession number: MT947598.2). The ITS2 sequence of *K. gibbosae* was extracted from the genome (accession number: PRJNA641212). We combined CO1 and ITS2 sequences and used two methods to perform phylogenetic analysis in PhyloSuite v1.2.2 (Zhang et al., 2020). First, we made multiple sequence alignments with MAFFT v7.036 (Katoh et al., 2019). According to the Akaike Information Criterion (AICc), PartitionFinder2 (Lanfear et al., 2017) revealed GTR + G as the best evolutionary model for our data (Guindon et al., 2010; Lanfear et al., 2012). Maximum likelihood (ML) tree was constructed using IQ-TREE (Nguyen et al., 2015). Second, we constructed a Bayesian tree (Ronquist et al., 2012) under GTR + G model (Guindon et al., 2010; Lanfear et al., 2012). Phylogenetic trees were visualized with FigTree v1.4.4^1^.

In order to elucidate the genetic relationships among various fig trees, a phylogenetic reconstruction based on three nuclear DNA markers, internal transcribed spacer (ITS), external transcribed spacer (ETS), and glyceraldehyde-3-phosphate dehydrogenase (G3pdh) was conducted. The sequences of three nuclear markers of the six species of fig trees corresponding to the six species of pollinators and the host of *K. gibbosae* (as outgroup) were obtained from NCBI, and the accession numbers were listed in Supplementary Table 2. The genetic distance over ITS-ETS-G3pdh sequences between fig trees was estimated with MEGA7 (Kimura, 1980; Kumar et al., 2016). The reconstruction process of ML (Nguyen et al., 2015) and Bayesian phylogenetic tree (Ronquist et al., 2012) was the same as described above, but under GTR + I + G, K81, TRN + I models or GTR + I + G, K80, F81 + I models for ITS, ETS, and G3pdh, respectively (Guindon et al., 2010; Lanfear et al., 2012, 2017). Then, the outgroup was removed and the phylogenetic tree was used for phylosymbiosis analysis.

### Molecular Species Delimitation

We carried out four approaches for species delimitation in order to have more robust results, including Automatic Barcode Gap Discovery (ABGD) (Puillandre et al., 2012), which detects the barcode gap based on the user-defined boundaries for intraspecific variability, then sorts the sequences into hypothetical species with *p*-values; a Java program uses an explicit, determinate algorithm to define Molecular Operational Taxonomic Unit (jMOTU) (Jones et al., 2011), and clustering-based approaches, e.g., Generalized Mixed Yule Coalescent (GMYC) (Pons et al., 2006), which uses likelihood to identify species boundaries by detecting the transition point between the speciation process and intraspecific lineage coalescence, and Bayesian implementation of the Poisson Tree Processes model (bPTP) (Zhang et al., 2013), which assumes independent exponential distributions to model the branch lengths for speciation and for coalescence. The first two methods (ABGD and jMOTU) were based on genetic distances, and the latter two methods (GMYC and bPTP) were based on the inferred tree (Pons et al., 2006; Jones et al., 2011; Puillandre et al., 2012; Zhang et al., 2013). The ABGD analyses were performed at the web server^2^, with the following settings: relative gap width *X* = 1.0, K2P distance and intraspecific divergence (*P*) values range from 0.001 to 0.1, and other parameter values employed defaults. The bPTP analyses were conducted on the web server^3^ using rooted phylogenetic input tree attached to Supplementary Figure 1, and the following settings were employed: 100,000 MCMC generations, thinning interval of 100, and the first 10% were discarded as burn-in. GMYC delimits distinct genetic clusters by optimizing the set of modes defining the transitions between inter- and intraspecific processes; the analysis was conducted using BEAST 1.8.0 under a strict clock model and speciation with Birth-Death process model (Drummond et al., 2012); the runs consisted of 10 million generations sampled every 1,000 cycles, convergence was assessed by ESS values, and a burn-in with 25% was set to obtain an optimal consensus tree; we then used the obtained tree to analyze the data under the GMYC species delimitation approach in the software R v4.0.1 (R Core Team, 2021) with the package *splits* using the single-threshold method (Ezard et al., 2015). The jMOTU uses predefined thresholds to calculate the genetic differences within average sequence length. The results of the species delimitation were visualized *via* iTOL v6^4^ (Letunic and Bork, 2021).

### 16S rDNA Library Preparation

The V4 region of the 16S rRNA gene was PCR-amplified at Novogene Bioinformatics Technology Co., Ltd. (Beijing, China) by specific primers (515F-806R) with the barcode following Kueneman et al. (2014). Each PCR reaction was carried out with 10 ng template DNA, 15 μl of Phusion^®^ High-Fidelity PCR Master Mix (New England Biolabs, Ipswich, MA, United States), and 2 μM of forward and reverse primers. One thermal cycle consisted of initial denaturation at 98°C for 1 min, followed by 30 cycles of denaturation at 98°C for 10 s, annealing at 50°C for 30 s, elongation at 72°C for 30 s, and finally 72°C for 5 min. Blank control was set during PCR amplification, and sterile water of equal volume was used instead of template.

Each sample was amplified, and the PCR products were tested for concentration using Qubit^@^ 2.0 Fluorometer (Thermo Fisher Scientific, Cleveland, OH, United States). There was only one sequencing library per sample. Equal concentrations of each sample were pooled, and the pooled amplicons were cleaned using Qiagen Gel Extraction Kit (Qiagen, Germany). Sequencing libraries were generated using TruSeq^®^ DNA PCR-Free Preparation Kit (Illumina, Inc., San Diego, CA, United States) following manufacturer’s protocols and index codes were added. The library quality was assessed on the Qubit^@^ 2.0 Fluorometer (Thermo Fisher Scientific, Cleveland, OH, United States) and Agilent Bioanalyzer 2100 system. Finally, the library was sequenced on Illumina NovaSeq6000 platform at the Novogene Bioinformatics Technology Co., Ltd. (Beijing, China) and 250 bp paired-end reads were obtained.

### Sequence Processing

The 54 sequencing libraries we obtained ranged in size from 30.4 to 57.1 M, with an average of 44.6 M. Paired-end reads were assigned to samples based on their unique barcode and truncated by cutting off the barcode and primer sequence. Paired-end reads were merged into a single sequence using Fast Length Adjustment of Short Reads (FLASH) v1.2.7, and the splicing sequences were called raw tags (Magoč and Salzberg, 2011). Then, raw tags were filtered and pre-processed in Qualitative Insights into Microbial Ecology (QIIME) v1.9.1 (Caporaso et al., 2010b; Bokulich et al., 2013). During this process, QIIME’s default quality-control parameters were used. The tags were compared with the Silva132 database using UCHIME algorithm to detect chimera sequences, and then all chimera sequences were removed (Edgar et al., 2011; Haas et al., 2011).

Sequence analyses were performed by Uparse v7.0.1001. Sequences with ≥ 97% similarity were assigned to the same operational taxonomic unit (OTU) (Edgar, 2013). Representative sequence for each OTU was screened for further annotation. The Silva132 database was used based on Mothur algorithm to annotate taxonomic information for each unique OTU (Quast et al., 2013).

### Data Analysis

The sequence number of the sample with the least sequences was used as the standard for normalization to obtain OTUs abundance information. Subsequent analyses of alpha diversity and beta diversity were all performed basing on this output normalized data.

The rarefaction curves, Venn diagram, and heat map of the top 20 classes in bacterial abundance were carried out in R v4.0.1 software (R Core Team, 2021). Then, we inferred phylogenies of the top 20 genera in bacterial abundance of fig wasp by FastTree (Price et al., 2009) based on the sequence alignments of Greengenes database (DeSantis et al., 2006) and PyNAST (Caporaso et al., 2010a) software, and the bacterial abundance information was added.

Observed species, Chao1, ACE, Shannon, Simpson, and PD whole tree were calculated for all samples as measures of alpha diversity. All these indices in our samples were obtained by QIIME v1.9.1 and visualized with R v4.0.1 software (Caporaso et al., 2010b; R Core Team, 2021). One-way ANOVA was performed to test the difference of alpha diversity indices among host populations using SPSS v24. Spearman method was used to examine the correlations between the proportion of *Wolbachia* and the Shannon, Simpson indices and performed by *ggpubr* package (Kassambara, 2020) in R v4.0.1 software (R Core Team, 2021) and functions in the package that comes with R itself. The significance of differences in alpha diversity indices between group *Wolbachia*-infected and non-infected was evaluated by *t*-test, and *t*-test was carried out in R v4.0.1 software (R Core Team, 2021). Before the *t*-test, function qqplot in *car* package (Fox and Weisberg, 2019) and function bartlett.test of R v4.0.1 (R Core Team, 2021) software were used for the test of normal distribution and variance homogeneity. The sample sizes of group *W*+ and *W*− were 36 and 18, respectively; the sample size of *C. solmsi*_1 or *C. solmsi*_2 was six. When two variables have equal variances, the two sample *t*-test was used; otherwise, the Welch two sample *t*-test was used (R Core Team, 2021).

Beta diversity was calculated based on weighted and unweighted Unifrac, Bray–Curtis, and binary Jaccard distances in QIIME v1.9.1 (Caporaso et al., 2010b). Non-Metric Multi-Dimensional Scaling (NMDS) analysis was performed to visualize complex, multidimensional data. In this study, NMDS analyses based on Bray–Curtis and weighted Unifrac were displayed by *vegan* package in R v4.0.1 software (Oksanen et al., 2020; R Core Team, 2021). In order to determine whether the inter-population differences were significantly different from those intra-population and to evaluate whether the grouping was meaningful, the significance test of inter-population differences using Analysis of Similarity (ANOSIM) based on the Bray–Curtis distance value was calculated. At the same time, Multi-Response Permutation Procedure (MRPP) parametric test based on Bray–Curtis was used to analyze whether there is significant difference in microbial community structure between populations. Both ANOSIM and MRPP conducted by *vegan* package in R v4.0.1 software (Oksanen et al., 2020; R Core Team, 2021). Unweighted Pair-group Method with Arithmetic Means (UPGMA) Clustering was performed as a type of hierarchical clustering method to interpret the distance matrix using average linkage and was conducted by QIIME v1.9.1 (Caporaso et al., 2010b).

The phylosymbiosis was validated using both matrix and topological comparisons. Mantel test was used to analyze the correlation between the genetic distance of fig pollinators or fig trees and the microbial beta diversity distance matrices with the *ade4* package in R v4.0.1 software (Dray and Dufour, 2007; R Core Team, 2021). In addition to the Mantel test, the Robinson–Foulds (Robinson and Foulds, 1981) and Matching Cluster (Bogdanowicz and Giaro, 2013) congruency analysis between the phylogenetic tree of pollinators or fig trees and microbial UPGMA cluster tree was carried out according to Brooks’ Python script (Brooks et al., 2016) and the TreeCmp program (Bogdanowicz et al., 2012). Statistical significance was evaluated by determining the probability of 100,000 randomized bifurcating dendrogram topologies with the same leaf nodes as the phylogenetic tree yielding equivalent or more congruent phylosymbiotic patterns than the microbiome dendrogram (Brooks et al., 2016). Normalized Robinson–Foulds (nRF) and normalized Matching Cluster (nMC) scores range from 0 (complete congruence) to 1.0 (complete incongruence) (Brooks et al., 2016).

## Results

### The Phylogeny of Insects and *Wolbachia* Infection

**FIGURE 1:**
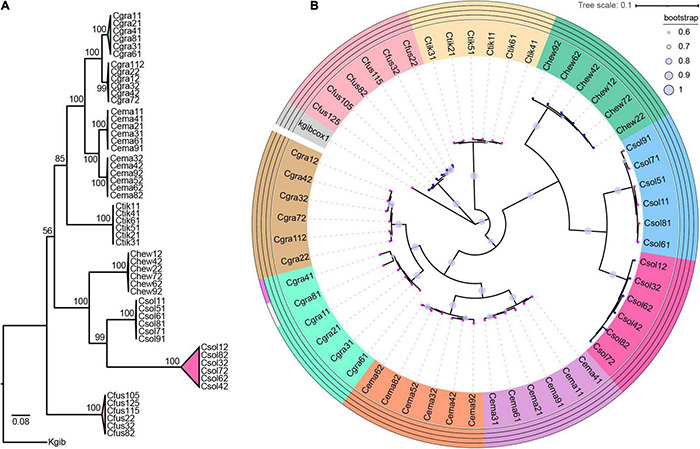
Host phylogeny and molecular species delimitation. **(A)** Phylogenies of *Ceratosolen* by ML method based on CO1 gene fragments and ITS2 sequences. **(B)** According to four methods of ABGD, jMOTU, bPTP, and GYMC, all the samples were defined as nine clades. Species delimitation results are shown in the outer circles. At the tip of the branch, the magenta pentagram indicates *Wolbachia*-infected, and the blue pentagram indicates uninfected. Fig wasp species are abbreviated as follows: Cgra, *Ceratosolen gravelyi*; Cfus, *Ceratosolen fusciceps*; Ctik, *Ceratosolen* sp.; Chew, *Ceratosolen hewitti*; Cema, *Ceratosolen emarginatus*; Csol, *Ceratosolen solmsi*; Kgib, *Kradibia gibbosae*.

After constructing the host phylogenetic tree by ML and Bayesian methods, we found that the same dendrogram was obtained from the two methods, and the phylogenetic relationship of all the species was well defined by CO1 genes combined with ITS2 sequences (Supplementary Figure 1). All the 54 samples were separated into nine clades, supported by a confidence value ranging from 56 to 100 (Figure 1A). Each of *C. gravelyi*, *C. emarginatus*, and *C. solmsi* had two clades. All conspecific individuals from the same population were clustered into one clade (Figure 1A and Supplementary Table 1).

Based on the approaches of ABGD, jMOTU, bPTP, and GYMC, all the samples were defined as nine clades, which was consistent with the results obtained from phylogenetic analysis (Figure 1). *C. gravelyi*, *C. emarginatus*, and *C. solmsi* were separated into two clades, although we did not find the morphological differences.

All the fig wasp individuals of *C. gravelyi*_1, *C. gravelyi*_2, *C. emarginatus*_1, *C. emarginatus*_2, *Ceratosolen* sp., and *C. solmsi*_1 were positive for *Wolbachia* infection (*W*+ group). No *Wolbachia* was detected using specific primers in the individuals from *C. fusciceps*, *C. hewitti*, and *C. solmsi*_2 (*W*− group) (Figure 1B).

### Overall Microbial Community Compositions of the Fig Pollinators

Overall, 54 samples were successfully examined. Bacterial communities’ compositions were studied using the Illumina NovaSeq6000 platform. The filtered, high-quality sequence database obtained was 3,478,098 sequences, which were classified into 13,331 unique OTUs by a cutoff of 97% sequence similarity. Rarefaction analysis was performed at a threshold of 3% sequences dissimilarity for all samples. The great majority of the samples reached an asymptote level, indicating that our sampling efforts were sufficient to obtain an accurate estimate of OTU richness (Supplementary Figure 2).

**FIGURE 2:**
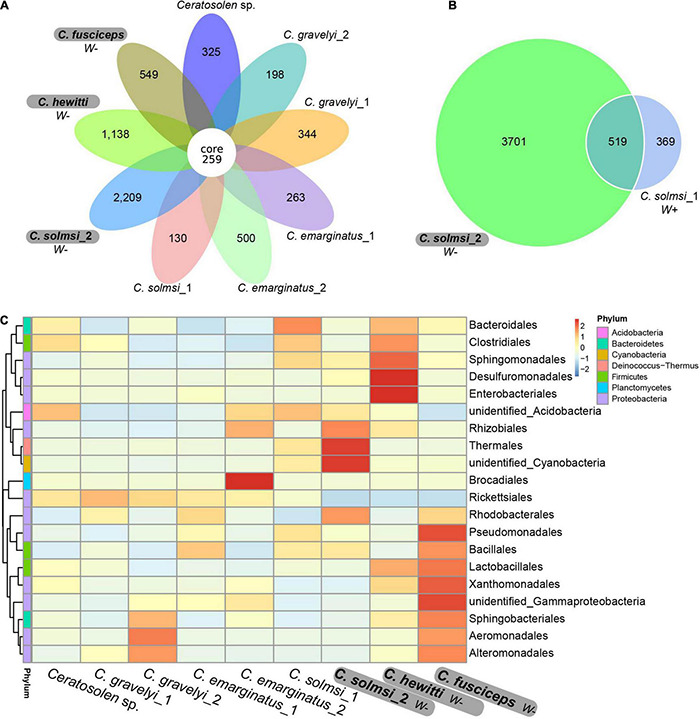
Venn diagram and heat map. **(A)** Venn diagram based on all nine population. **(B)** Venn diagram between *Wolbachia* infected and uninfected group of *C. solmsi*. **(C)** Heat map of the top 20 classes in bacterial abundance. Three populations in bold are free of *Wolbachia* (*W*–), while the other six populations are infected with *Wolbachia* (*W*+).

The Venn diagram showed the unique and shared core OTUs among the populations. Of the total 13,331 OTUs obtained, 259 OTUs were shared by nine populations (Figure 2A). The proportions of shared OTUs in populations of *W*− group were lower than that of *W*+ group. Correspondingly, the populations in *W*− group had more specific OTUs than the populations in *W*+ group. In particular, the specific OTUs of *C. solmsi* in the *W*− group were almost 17 times higher than that in the *W*+ group (Figure 2B). A similar trend was also shown in the heat map of the top 20 classes in bacterial abundance, with more hot areas in the populations of *W*− group (Figure 2C).

**FIGURE 3:**
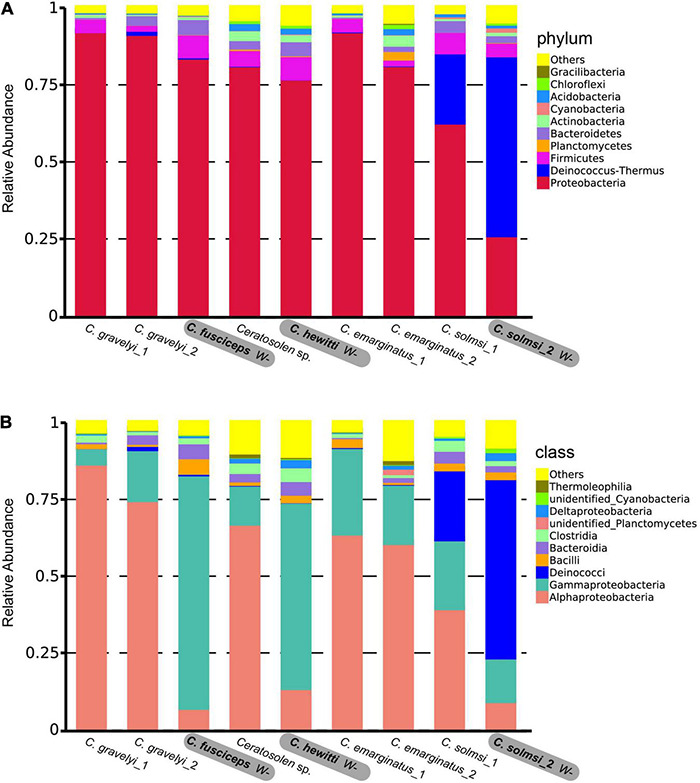
Microbial community composition. Relative abundance of bacteria at the level of phylum **(A)** and class **(B)** in nine populations of fig wasp. Three populations in bold are free of *Wolbachia* (*W*–), while the other six populations are infected with *Wolbachia* (*W*+).

In total, 12,763 (95.74%) OTUs were annotated based on Silva132 database. Approximately 95.75, 72.86, 61.20, 51.38, 29.80, and 8.12% of the OTUs were annotated at the level of phylum, class, order, family, genus, and species, respectively. Overall, 79 different phyla were detected. At the phylum level, the composition of bacteria was similar in all *Ceratosolen* samples, with Proteobacteria being the predominant phylum for all the populations except for *C. solmsi_*2 (Figure 3A). The subsequent dominant phyla were Firmicutes and Bacteroidetes. In the case of *C. solmsi_*2, Deinococcus-Thermus was dominant and followed by Proteobacteria, Firmicutes, and Bacteroidetes. At the class level, the populations from the *W*+ group were dominated by Alphaproteobacteria, while the populations from the *W*− group except for *C. solmsi*_2 were dominated by Gammaproteobacteria (Figure 3B). At the genus level, the dominant genus identified was *Wolbachia* for the *Wolbachia*-infected populations (Supplementary Figure 3), at a prevalence of 20.3–96.4%. By contrast, the dominant genus in *C. fuscipes*, *C. hewitti*, or *C. solmosi*_2 was totally different. It was *Acinetobacter*, *Citrobacter*, or *Thermus*, respectively. Surprisingly, almost half of the sequences could not be identified at the level of genera in *C. hewitti*.

In our study, a total of nine unique OTUs were classified as *Wolbachia*. In detail, OTU1 (93.5%) and OTU8 (6.2%) had higher abundance and totally accounted for 99.7% of OTUs classified as *Wolbachia* in *W*+ group. In *C. gravelyi*_1, OTU1 and OTU8 accounted for 86.8 and 13.0% of OTUs classified as *Wolbachia*, respectively. Similarly, OTU1 and OTU8 accounted for 83.3 and 16.3% of OTUs classified as *Wolbachia* in *C. gravelyi*_2. As for the other four *W*+ populations, OTU1 accounted for more than 98.9% of the OTUs classified as *Wolbachia*.

### Similarity of Bacterial Communities Within and Between Fig Pollinator Species

We calculated and compared alpha diversity indices of the fig wasp microbiota composition at the OTU level, and the variations of microbiota between and within various populations were shown in Table 1. Among all the populations, *C. hewitti* had the highest bacterial richness indices, while *C. solmsi*_1 had the lowest. *C. solmsi*_2 had the greatest variability.

**TABLE 1:** Alpha diversity indices and percentages of shared OTUs among populations.

	Richness estimates			Diversity estimates			Core microbiota
Population	Observed species	Chao 1	ACE	Shannon	Simpson	PD whole tree	Shared OTUs (%)
*C. hewitti*	2613 ± 975^a^	2994 ± 1032^a^	3236 ± 1061^a^	5.44 ± 2.64^a^	0.742 ± 0.292^ab^	230.8 ± 70.5^a^	18.54
*Ceratosolen* sp.	2259 ± 748^a^	2681 ± 834^a^	2940 ± 891^a^	3.94 ± 1.34^a^	0.567 ± 0.160^bc^	187.1 ± 36.9^abc^	44.35
*C. fusciceps*	1239 ± 325^a^	1420 ± 349^a^	1536 ± 364^a^	4.38 ± 1.25^a^	0.777 ± 0.119^a^	195.2 ± 57.4^abc^	32.05
*C. emarginatus*_2	1901 ± 769^ab^	2233 ± 836^a^	2454 ± 891^a^	3.91 ± 1.10^a^	0.640 ± 0.084^abc^	198.4 ± 35.8^ab^	34.12
*C. emarginatus*_1	1017 ± 344^ab^	1290 ± 464^ab^	1473 ± 567^ab^	2.73 ± 0.61^a^	0.564 ± 0.146^bc^	142.6 ± 53.0^cd^	49.62
*C. gravelyi*_1	1033 ± 427^ab^	1211 ± 468^ab^	1340 ± 490^ab^	2.41 ± 1.10^a^	0.456 ± 0.137^c^	95.8 ± 24.8^d^	42.95
*C. gravelyi*_2	728 ± 486^ab^	858 ± 556^ab^	949 ± 603^ab^	2.66 ± 0.82^a^	0.577 ± 0.109^bc^	92.3 ± 27.6^d^	56.67
*C. solmsi*_2	1280 ± 1115^ab^	1412 ± 1233^ab^	1503 ± 1314^ab^	4.30 ± 2.66^a^	0.722 ± 0.215^ab^	160.1 ± 69.1^bc^	10.49
*C. solmsi*_1	456 ± 47^b^	497 ± 50^b^	521 ± 51^b^	3.93 ± 0.42^a^	0.809 ± 0.091^a^	101.2 ± 28.7^d^	66.58

*The values of alpha diversity indices are expressed as mean ± SD. One-way ANOVA was used to compare the differences of alpha diversity indices among populations. Significant differences are indicated by different letters (P < 0.05) in the same column.*

**FIGURE 4:**
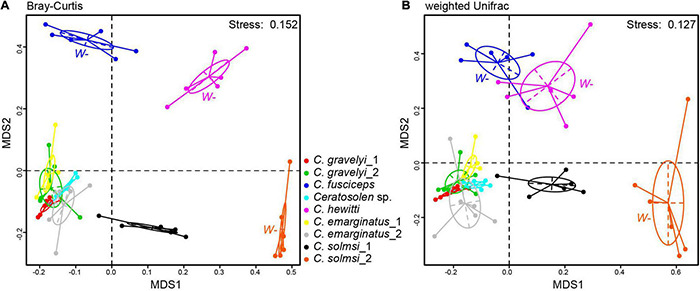
Microbial community structure. NMDS ordinations of fig wasp microbiota based on Bray–Curtis (stress = 0.152) **(A)** and weighted Unifrac (stress = 0.127) dissimilarity matrices **(B)** on OTU level. Each dot represents a sample. Fig wasps from the same populations are colored uniformly. Three populations that are not infected with *Wolbachia* are labeled *W*–, while the other six populations are infected. Ellipses = 80% confidence intervals.

The NMDS analysis was performed on the bacterial compositions of nine populations of fig wasps. The global differences in microbial community compositions were clearly visualized in the NMDS plots based on Bray–Curtis and weighted UniFrac distance metrics (Figure 4). The individuals of the same population clustered together. ANOSIM revealed that each population harbored a unique bacterial community composition (*P* < 0.05, *R* > 0) (Supplementary Table 3). Consistently, MRPP analysis confirmed the significant differences in bacterial community composition among various populations (*P* < 0.05, *A* > 0) (Supplementary Table 3).

In addition, the distribution of *Wolbachia*-infected samples was relatively concentrated in NMDS analysis, which should be caused by the high abundance of *Wolbachia* (Figure 4). The populations from *W*+ group were concentrated, and close to each other (Figure 4). The populations from *W*− group were scattered, and the bacterial community structures were quite different from each other. On the whole, the *W*− group was deviated from the *W*+ group.

### Effects of *Wolbachia* on the Microbial Communities

**FIGURE 5:**
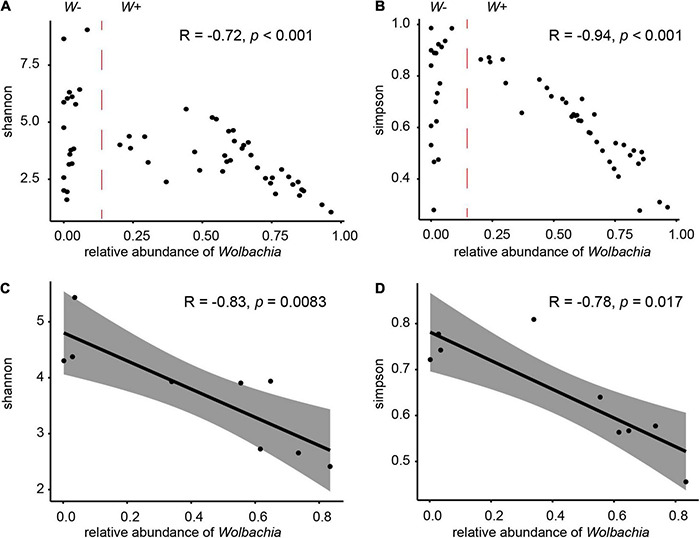
Effects of *Wolbachia* on the microbial communities. Relationships between the proportions of *Wolbachia* and the Shannon indices **(A)** and Simpson indices **(B)** of microbial community in samples from group *W*+. Relationships between the proportions of *Wolbachia* and the Shannon indices **(C)** or Simpson indices **(D)** of microbial community in nine populations. *R* values and *P*-values of Spearman’s rank correlation are provided.

Spearman’s rank correlation analyses showed that the proportion of *Wolbachia* was significantly negatively correlated with the Shannon (*r* = − 0.72, *P* < 0.001) and Simpson (*r* = −0.94, *P* < 0.001) indices across all *W*+ samples (Figures 5A,B). For samples not infected with *Wolbachia* (*W*− group, the relative abundance of *Wolbachia* was close to zero), the effect of *Wolbachia* on the diversity of microbial communities was not counted. When the analysis was carried out by population, the populations that were not infected with *Wolbachia* tended to have a more diverse microbial community compared with the populations that were infected, and with the presence of *Wolbachia* and the increase in relative abundance, a significant negative correlation between the relative abundance of *Wolbachia* and diversity index was also observed at the population level (Figures 5C,D). We also examined whether there were significant differences in the diversity index of the microbiota between the *Wolbachia*-infected and uninfected samples. The Shannon and Simpson indices of group *W*+ and *W*−, *C. solmsi*_1, and *C. solmsi*_2 conformed to the normal distribution; the Simpson indices of group *W*+ and *W*− (*P* = 0.1858) and *C. solmsi*_1 and *C. solmsi*_2 (*P* = 0.0849) had equal variances, while the Shannon indices of group *W*+ and *W*− (*P* = 0.0007) and *C. solmsi*_1 and *C. solmsi*_2 (*P* = 0.0011) did not have equal variances. According to the *t*-test, there were significant differences between *W*+ and *W*− groups in Shannon (*P* = 0.016) and Simpson (*P* = 0.006) indices. However, there was no significant difference between *C. solmsi*_1 and *C. solmsi*_2 in Shannon (*P* = 0.75) and Simpson (*P* = 0.38) indices.

### Phylosymbiosis Between Fig Pollinators or Fig Trees and Microbiota

**FIGURE 6:**
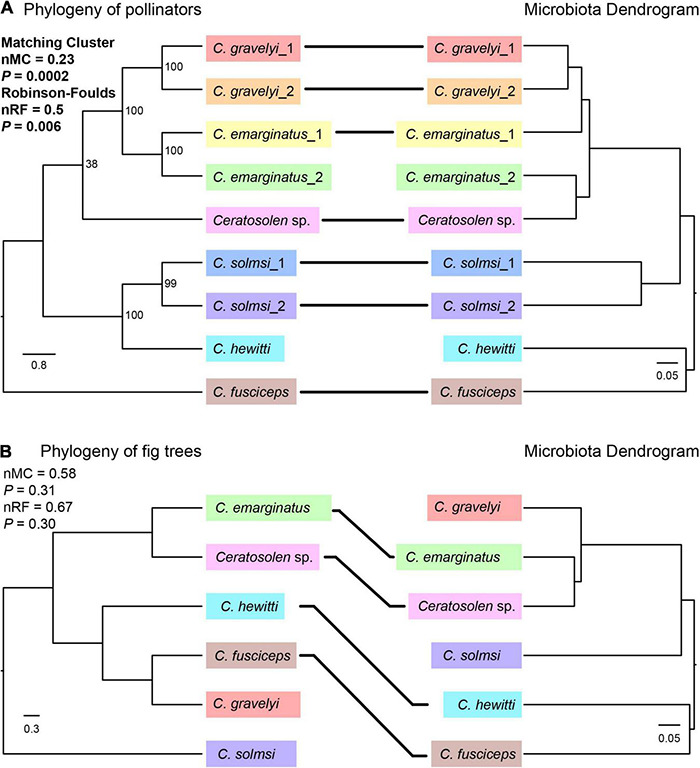
Phylosymbiosis test between the phylogeny of pollinators **(A)** or fig trees **(B)** and microbiota dendrogram. The phylogeny of pollinators was based on CO1 and ITS2 sequences by ML method. The phylogeny of fig trees was based on ITS, ETS, and G3pdh genes by Bayesian method. For the convenience of comparison, the names of pollinators were used instead of the names of fig trees in the phylogenetic tree. Microbiota composition dendrogram was generated by the Bray–Curtis matrix. The nRF and nMC scores range from 0.0 (complete congruence) to 1.0 (complete incongruence). Solid lines connect species whose position is concordant between the phylogeny of pollinators or fig trees and microbiota dendrogram.

To examine whether the *Ceratosolen* fig wasp phylogeny and their bacterial communities followed patterns of phylosymbiosis, we used both matrix comparisons and topological comparisons to quantify the signal of phylosymbiosis. Based on the genetic matrix of insect hosts and the beta diversity distance matrices, Mantel tests showed significant patterns of phylosymbiosis as measured by Bray–Curtis (*r* = 0.61, *P* = 0.008), weighted Unifrac (*r* = 0.73, *P* = 0.003), and unweighted Unifrac (*r* = 0.55, *P* = 0.005) distance metrics, while no significant pattern as measured by binary Jaccard (*r* = 0.42, *P* = 0.066) distance metric. Both the Robinson–Foulds and matching cluster metrics were used to detect phylosymbiosis between the microbiota dendrogram and their hosts’ phylogenetic tree. We observed significant phylosymbiosis signals with Bray–Curtis distances, although the microbiota dendrogram and their hosts’ phylogenetic tree were not completely congruent. More specifically, we found statistically significant congruence between fig wasp phylogeny and microbiota dendrograms using normalized Robinson–Foulds (nRF = 0.5, *P* = 0.006) and the matching cluster method (nMC = 0.23, *P* = 0.0002) (Figure 6A).

In order to determine whether the phylogeny of fig tree has an effect on the microbiota of fig pollinators, we examined whether there were phylosymbiotic signals between the fig trees and microbiota of fig pollinators based on both matrix comparisons and topological comparisons. Mantel tests showed no significant patterns of phylosymbiosis as measured by Bray–Curtis (*r* = 0.22, *P* = 0.15), weighted Unifrac (*r* = 0.19, *P* = 0.18), unweighted Unifrac (*r* = 0.14, *P* = 0.32), and binary Jaccard (*r* = 0.19, *P* = 0.26) distance metrics. Topological comparisons also showed that there were no significant phylosymbiotic signals between the fig pollinators’ microbiota and the fig trees (nRF = 0.67, *P* = 0.30; nMC = 0.58, *P* = 0.31), although four of the six species shared the same topological location (Figure 6B).

## Discussion

In this study, we use the fig pollinators of the genus *Ceratosolen* to explore the effects of the phylogenetic relationship of fig wasp hosts, the endosymbiont *Wolbachia*, and the unique symbiotic environment provided by fig fruit on the host bacterial communities. Our results show significant phylosymbiosis signals between fig pollinators and their microbiota, regardless of matrix comparisons or topological comparisons. In the analysis of phylosymbiosis, we detect statistically significant congruence between fig wasp phylogeny and microbiota dendrogram, either using the Robinson–Foulds (RF) metric or the modified matching cluster (MC) method which better accounts for sections of subtree congruence (Robinson and Foulds, 1981; Bogdanowicz and Giaro, 2013). These results indicate that the microbial communities of fig wasps in genus *Ceratosolen* are not formed randomly, but have a certain correlation with the host phylogeny.

Phylosymbiosis means the consistent relationship between hosts’ phylogeny and their microbial community structures. Due to that the fig pollinators spend most of their lives in the enclosed environment provided by fig fruits, and their food source is provided steadily by the ovaries of female flowers, they are much less likely to be disturbed by external environment than those living freely in open environment. Under this circumstance, the closely related fig pollinators species within the same genus are speculated to have similar microbial community structure. However, our study shows that the microbial community structures are distinguishable among the *Ceratosolen* species. The differences in microbial compositions can reflect the phylogeny of the hosts, suggesting that host phylogeny plays a non-negligible role in shaping host-associated microbial communities. From another perspective, the microbial community may play a promoting/delaying role in host speciation. Hosts are better adapted to their native microbiota than to foreign ones (Brooks et al., 2016; Parker et al., 2018). For instance, transplanting interspecific microbial communities in *Peromyscus* deer mice significantly decreased their ability to digest food, and *Nasonia* wasps that received transplants of microbial communities from different wasp species had lower survival rate than those given their own microbiota (Brooks et al., 2016). In *Nasonia* parasitoid wasps, the microbiome shifts in hybrids, as a rare *Proteus* bacteria become dominant, so the larval hybrids then catastrophically succumb to bacterium-assisted lethality and reproductive isolation between the species (Cross et al., 2021). All these data showed that host characteristics could regulate microbial communities, and changes in the microbial communities were involved in host hybridization lethality, which reflected a mutually restrictive coevolutionary relationship between the hosts and associated microbiota. Therefore, the phylosymbiosis signals found in the *Ceratosolen* fig pollinators, on the one hand, indicate that different species of fig wasps have formed their own stable microbial communities during the process of systematic differentiation, and on the other hand, they also imply that the microbial community may play an important role in the speciation of closely related fig pollinators.

Although these fig pollinators we used have a close relationship within the same genus, and the symbiotic environment and food sources provided by various fig fruits are similar, the particular symbiosis environment provided by fig fruits may have played an important role in shaping the different microbial communities of these closely related pollinators. The fig pollinators used in this study are associated to six different fig tree species. The fruits seem similar, but we cannot completely conclude that the fruits of different fig species provide the same source of nutrients for the fig pollinators living within them. Therefore, we cannot rule out the influence of diets on the bacterial community of these fig pollinators. There are few reports on bacterial communities associated with plant reproductive organs. Akduman et al. (2018) isolated and cultured bacteria of figs from *Ficus mauritiana* (La Re’union), *Ficus racemosa* (Vietnam), and *Ficus sycomorus* (South Africa), and isolated strains belonging to four bacterial phyla, Proteobacteria, Firmicutes, Bacteriodetes, and Actinobacteria. In our study, the bacterial communities of fig pollinators are mainly distributed in Proteobacteria, Firmicutes, and Bacteriodetes (Figure 3A), which is similar to the gut microbiota of other insects, such as *Nasonia* wasps, mosquitoes, and *Drosophila* flies (Brooks et al., 2016). Up to now, there are no studies that clearly explain the consistent relationship between figs and fig pollinators. However, there is a report that fungal communities in syconia and on pollinating wasps were similar (Martinson et al., 2012). Another study has shown that host phylogeny shapes the foliar endophytic fungal assemblages of *Ficus* (Liu et al., 2019). Fig pollinators only feed on fig fruits (Janzen, 1979) and carry the microbiota of their environment which is dictated by the evolution of the fig trees, but the fig trees do not show a phylosymbiotic pattern with the microbiota inside the pollinators (Figure 6B), thus do not fully dictate the evolution of the association wasp-microbiota. As have been shown in herbivorous rodents, although diet and geography had an impact on the structure of the natural microbiota, the effects of host phylogeny were stronger for both wild and captive animals (Weinstein et al., 2021), indicating that host genetic background is the most significant predictor of microbiota composition and stability than geography and diet in woodrats. At the same time, the enclosed living environment provided by fruits also naturally provides an opportunity for ecological space isolation for different fig pollinators to a large extent. In addition, sampling time or locations of different populations are diverse, which also provides an isolated environment in time or space. Therefore, the differences in nutritional conditions between populations and the temporal and spatial isolation may have played an important role in shaping the microbial community structures of these fig pollinators. The study of phylosymbiosis between fig pollinators and their associated microbiota would be more convincing when wasps are collected at the same site to avoid covariation, such as environmental (temperature, humidity, and so on), and under more strictly controlled conditions such as sex or nutritional variables that may introduce distortions in the analysis of the microbial community. However, the distinguishability of microbial communities caused by these factors should be a random separation pattern between populations, and the successful detection of phylosymbiosis signals indicates that the microbial community structures of fig pollinators may be mainly associated to the host phylogeny.

Our study provides the first thorough characterization of microbiota in fig wasps. Six of nine fig pollinator populations were infected with the intracellular symbiotic *Wolbachia*. *Wolbachia* as the dominant genera identified accounted for 61.96% of the whole bacterial community in the infected fig wasp populations, which was concordant with that *Wolbachia* was the dominant genus in Hymenoptera (Yun et al., 2014). Some *Wolbachia*-infected beetles and terrestrial isopods also showed high abundance of *Wolbachia* (Dittmer et al., 2014, 2016; Parker et al., 2020). The high abundance of *Wolbachia* in multiple hosts reminds us that endosymbionts are considerable factors shaping microbial diversity in the hosts. Despite *Wolbachia* have a widespread distribution in insects, little is known about how *Wolbachia* interacts directly with other bacteria within hosts. Our results suggest that *Wolbachia* can have a negative effect on the bacterial diversity of the fig pollinators. We find that the populations of *W*+ group have a relatively high proportion of core OTUs, which is consistent with that the relative abundance of *Wolbachia* is significantly negatively correlated with the diversity indices across all populations. These results indicate that infection with *Wolbachia* can lead to a decrease in the bacterial diversity of fig wasps. In natural *Drosophila* populations, it was also showed that the microbiota composition varied significantly with the relative abundance of *Wolbachia* (Fromont et al., 2019). Other similar results have been found in the small brown planthopper that *Wolbachia* infection, for instance, appears to play a greater role in shaping the microbial community structure than abiotic factors, resulting in a sharp decline in the diversity and abundance of host bacterial taxa (Duan et al., 2020). These studies suggest that *Wolbachia* does have an impact on the bacterial diversity of insect hosts and the underlying mechanism deserves investigation.

In this study, *C. gravelyi*, *C. emarginatus*, and *C. solmsi* are discovered to have cryptic species based on distinct mitochondrial derived DNA barcode CO1, nuclear DNA marker ITS2 sequences, and indistinguishable morphological characteristics. We here thus explore the underlying reasons from the perspective of collection locations and microbiota community. First, *C. emarginatus*_1 and *C. emarginatus*_2 were collected from Guangxi and Hainan provinces, respectively, and both collection sites are about 400 km apart and separated by the Qiongzhou Strait. *C. solmsi*_1 and *C. solmsi*_2 were collected from Guangdong and Hainan provinces, respectively, and both collection sites are about 500 km apart and separated by the Qiongzhou Strait as well. Therefore, geographical isolation may be related to the differentiation between both pairs of cryptic species. Second, in the composition of microbiota, *C. solmsi*_1 is infected with *Wolbachia*, while *C. solmsi*_2 is not, and NMDS analysis can distinguish the microbial communities of *C. solmsi*_1 and *C. solmsi*_2 well. As intracellular symbiotic bacteria that widely infect insect species and can perform reproductive manipulation on the hosts (Hertig and Wolbach, 1924; Hilgenboecker et al., 2008; Werren et al., 2008), *Wolbachia* has been predicted to be the internal driving force for reproductive isolation (Bordenstein et al., 2001), which may also be the putative reason for the differentiation of the cryptic species of the *C. solmsi*. Besides, in the species of *C. gravelyi*, although both populations of *C. gravelyi*_1 and *C. gravelyi*_2 were collected from the Xishuangbanna Tropical Botanical Garden (Yunnan Province, China), *C. gravelyi*_1 has more unique microbial strains in terms of the proportion of the core OTUs of the microbial community. As for bacterial abundance, *C. gravelyi*_1 and *C. gravelyi*_2 have significant difference (*t*-test, *P* < 0.05, the same below) only in the phylum of Bacteroidetes, while between *C. emarginatus*_1 and *C. emarginatus*_2, and between *C. solmsi*_1 and *C. solmsi*_2, there are significant differences in Proteobacteria and some other bacterial phyla. Among the core bacteria (Supplementary Table 4), we find significant differences in the abundance of the order of Pseudomonadales and the genus of *Acinetobacter* between each of the three pairs of cryptic species. Many bacteria belonging to *Acinetobacter* are nosocomial pathogens (Bergogne-Bérézin and Towner, 1996), but their physiological roles in insects are still unclear. Changes in the abundance of core microbiota may affect host fitness and thus contribute to their evolutionary divergence (Rosenberg et al., 2010). Therefore, besides the geographical isolation, the intracellular symbiotic *Wolbachia* or other microbial components may also play a role in the divergence of the cryptic species of the three fig pollinator species.

Interestingly, our high-throughput 16S rDNA microbial community profiles reveal that these fig pollinators may have been infected with a variety of intracellular symbiotic bacteria in their evolutionary history. In our survey of *Wolbachia* infection in the fig pollinators, the individual was considered to be uninfected if all three pairs of *Wolbachia*-specific primers failed to amplify *Wolbachia*-specific gene. In the final results, the OTUs classified as *Wolbachia* are also observed as a rare microbial community member in the *W*− populations, including *C. fusciceps* (2.9%), *C. hewitti* (3.6%), and *C. solmsi*_2 (0.2%). While in the six *W*+ populations, *Wolbachia* is detected at high abundance with relative abundance ranging from 20.3 to 96.4%. Due to that few OTUs belonging to *Wolbachia* were found in *W*− group, we speculate that these samples may contain footprints of historic *Wolbachia* infections that mostly have been lost, or these samples acquired a small amount of *Wolbachia* through horizontal transmission recently. In addition to *Wolbachia*, reads of some other intracellular symbiotic bacteria, such as *Buchnera*, *Spiroplasma*, *Arsenophonus*, *Blattabacterium*, *Rickettsiella*, *Serratia*, and *Candidatus Fritschea*, have also been detected in our samples, even though the relative sequence abundances of these bacteria are all lower than 0.01%. Excluding the possibility of contamination, these findings can provide evidences for the existence of multiple intracellular symbionts in the fig pollinators. Another very surprising finding is the phylum of Deinococcus-thermus, which has an abundance as high as 22.9 and 58.6% in *C. solmsi*_1 and *C. solmsi*_2, respectively, but the abundance in other populations is extremely low. Deinococcus-Thermus is considered as extremophiles bacteria (Griffiths and Gupta, 2007) and can be found in the gut of insects (Yun et al., 2014). It needs more work to explore why such a high proportion of Deinococcus-Thermus is present in the *C. solmsi* samples.

## Conclusion

In this study, a phylosymbiosis signal was found between fig wasps of genus *Ceratosolen* and their microbial community, which help us to view the role of host phylogeny in shaping microbial community structure from a fundamental perspective. The presence of *Wolbachia* led to a decrease in host microbial diversity, and *Wolbachia* seemed to be the dominant species in the infected hosts. Our studies demonstrated that host phylogeny and the presence of endosymbiotic *Wolbachia* were the driving factors of bacterial community structure in *Ceratosolen* fig wasps. Collectively, the tripartite interactions of host, symbionts, and microbiota shape the dynamic stability of the holobiont ecosystem in fig wasps.

## Data Availability Statement

The datasets presented in this study can be found in online repositories. The names of the repository/repositories and accession number(s) can be found below: https://figshare.com/, 10.6084/m9.figshare.16853656, 10.6084/m9.figshare.17078801, and 10.6084/m9.figshare.17075978.

## Author Contributions

JX and DH designed the project. JL and XW collected the fig wasp samples and completed data analyses. JL completed laboratory work. JL, JX, and XW wrote the manuscript. DH revised the manuscript. All authors have approved the final manuscript.

## Conflict of Interest

The authors declare that the research was conducted in the absence of any commercial or financial relationships that could be construed as a potential conflict of interest.

## Publisher’s Note

All claims expressed in this article are solely those of the authors and do not necessarily represent those of their affiliated organizations, or those of the publisher, the editors and the reviewers. Any product that may be evaluated in this article, or claim that may be made by its manufacturer, is not guaranteed or endorsed by the publisher.

## Footnotes

1
http://tree.bio.ed.ac.uk/software/figtree/

2
https://bioinfo.mnhn.fr/abi/public/abgd/abgdweb.html

3
https://species.h-its.org

4
https://itol.embl.de
