# Supplementary figures and images for: The Phylosymbiosis Pattern Between the Fig Wasps of the Same Genus and Their Associated Microbiota
Source: Front Microbiol. 2022 Feb 14;12:800190. doi: 10.3389/fmicb.2021.800190 (PMC8882959; doi:10.3389/fmicb.2021.800190)

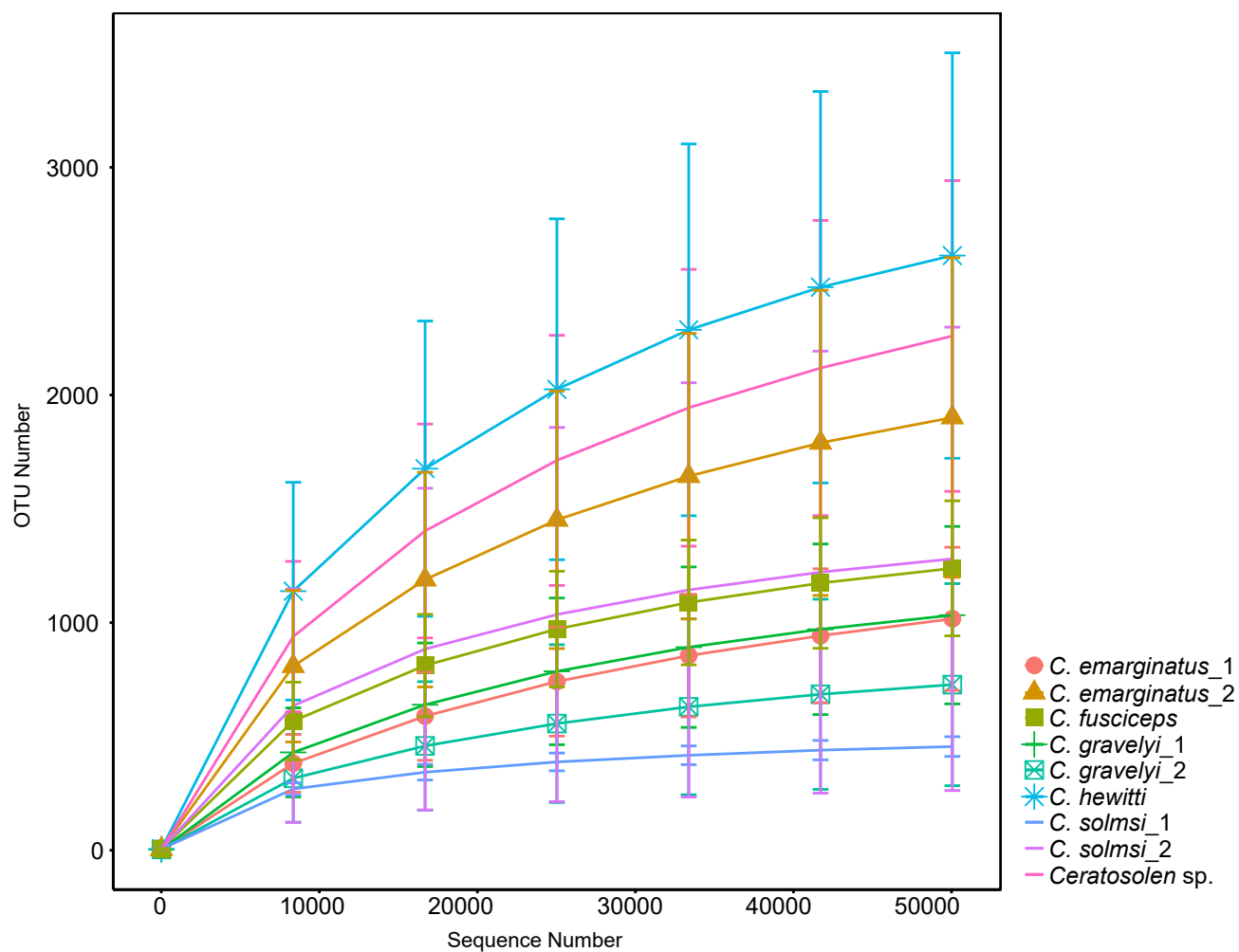

**Supplementary Figure S2:** Rarefaction curves of nine populations of fig wasp.

Supplement: Supplementary file 5 [file Data_Sheet_1.PDF]
