# Supplementary material for: The Phylosymbiosis Pattern Between the Fig Wasps of the Same Genus and Their Associated Microbiota
Source: Front Microbiol. 2022 Feb 14;12:800190. doi: 10.3389/fmicb.2021.800190 (PMC8882959; doi:10.3389/fmicb.2021.800190)
Supplement: Supplementary file 6 [file Data_Sheet_2.PDF]

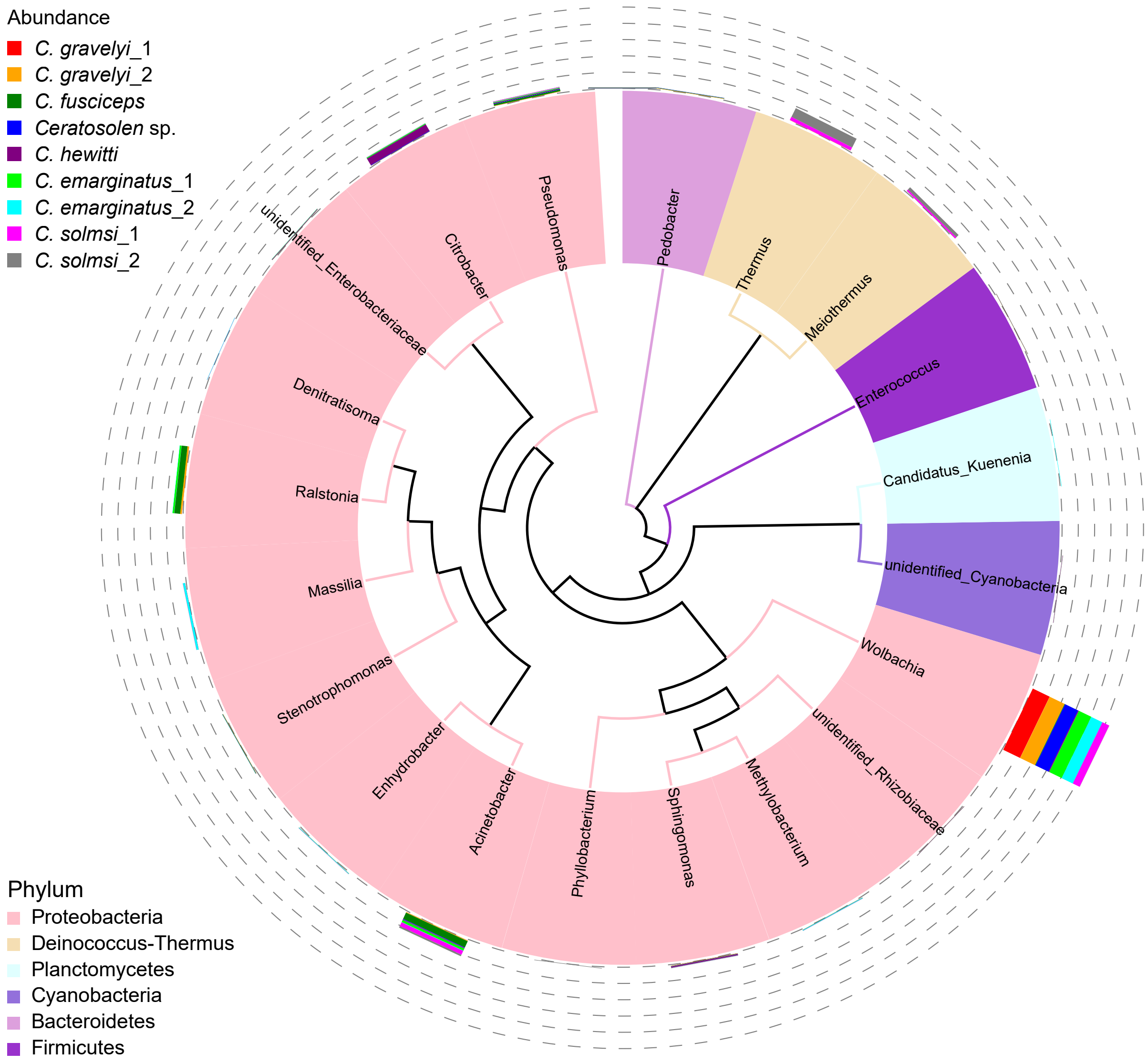

Supplementary Figure S3: Phylogenies of the top 20 genera in bacterial abundance of fig wasp, and the bacterial abundance information was provided.
