# Supplementary material for: The Phylosymbiosis Pattern Between the Fig Wasps of the Same Genus and Their Associated Microbiota
Source: Front Microbiol. 2022 Feb 14;12:800190. doi: 10.3389/fmicb.2021.800190 (PMC8882959; doi:10.3389/fmicb.2021.800190)
Supplement: Supplementary file 7 [file Data_Sheet_3.PDF]

A: IQ-TREE

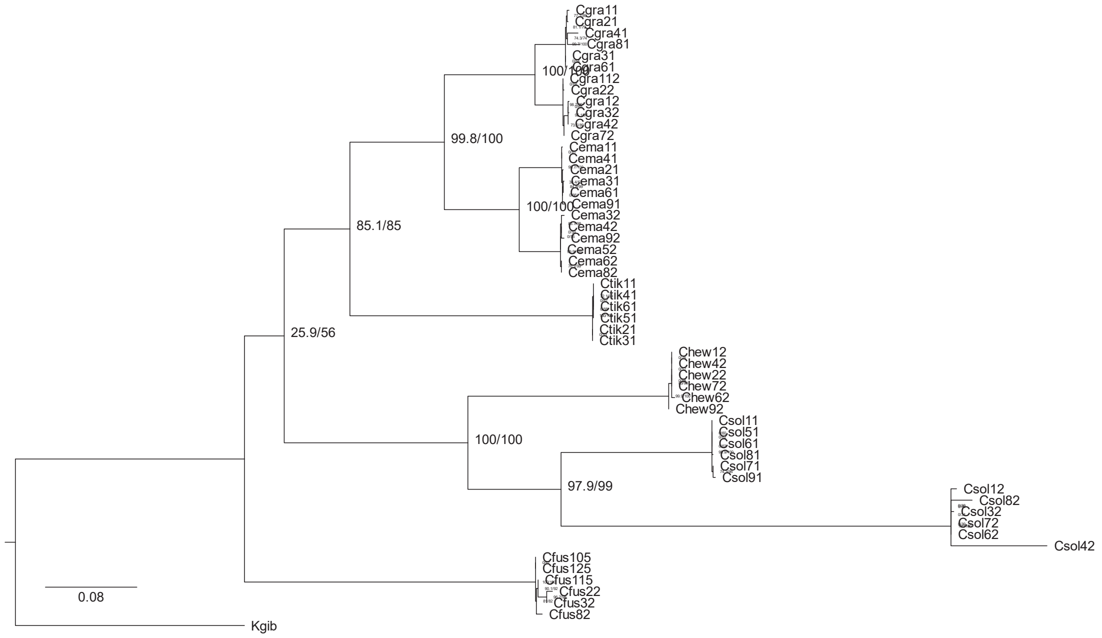

B: BI-TREE

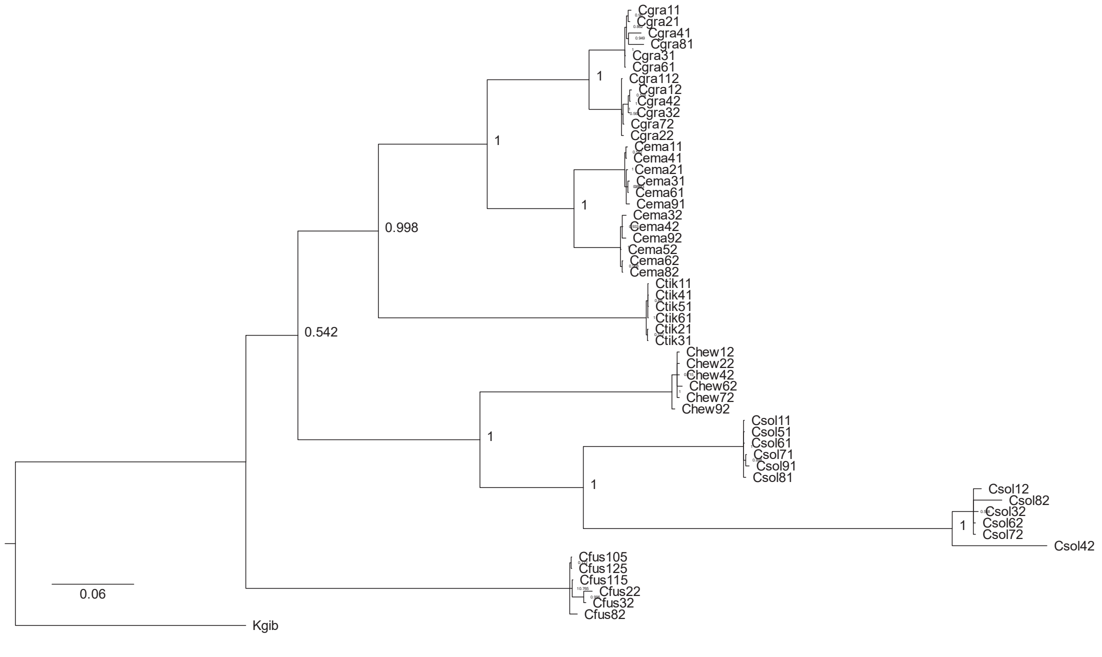

Supplementary Figure S1: Phylogenetic tree of fig wasps within genus *Ceratosolen* by ML (A) and Bayesian methods (B) based on CO1 and ITS2 sequences. Fig wasp species are abbreviated as follows: Cgra, *Ceratosolen gravelyi*; Cfus, *Ceratosolen fusciceps*; Ctik, *Ceratosolen* sp.; Chew, *Ceratosolen hewitti*; Cema, *Ceratosolen emarginatus*; Csol, *Ceratosolen solmsi*; Kgib, *Kradibia gibbosae*.

C

IQ-TREE

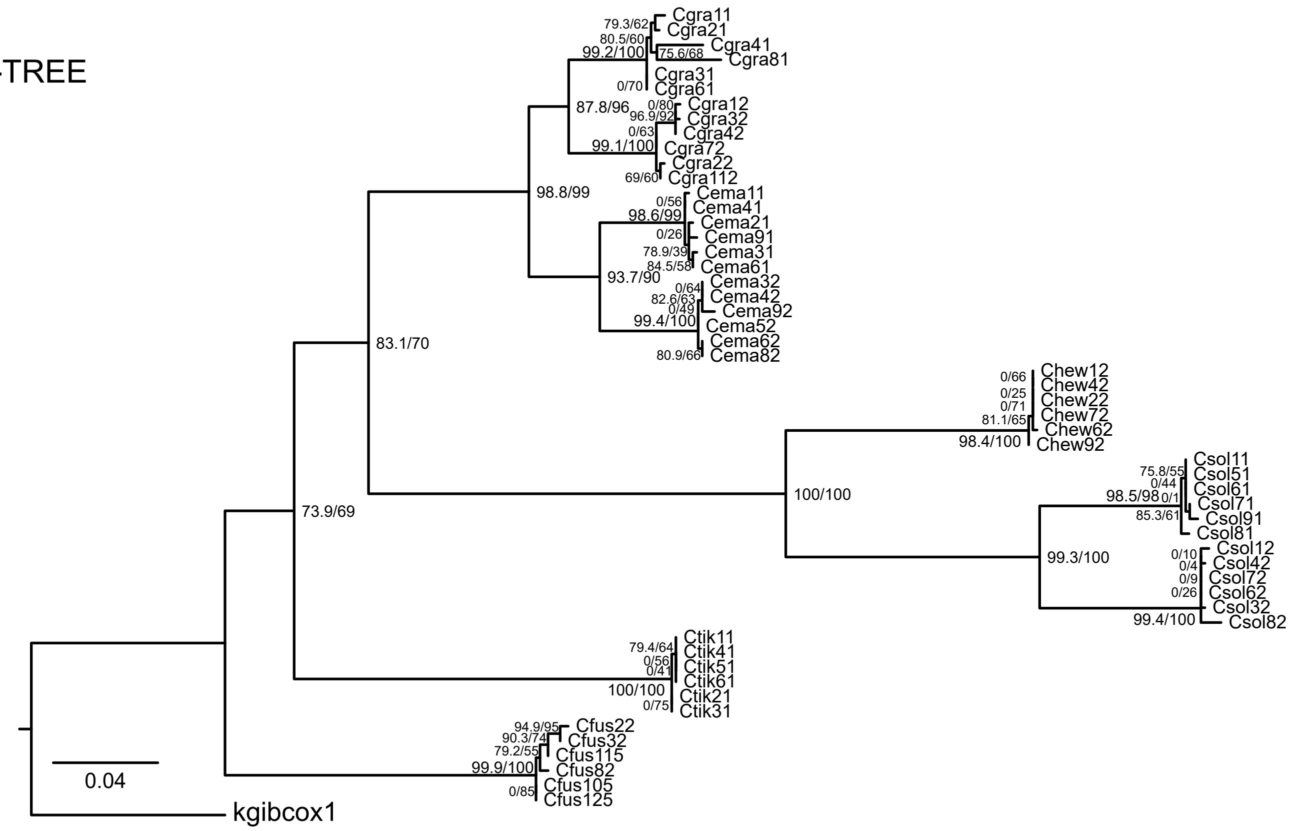

D

BI-TREE

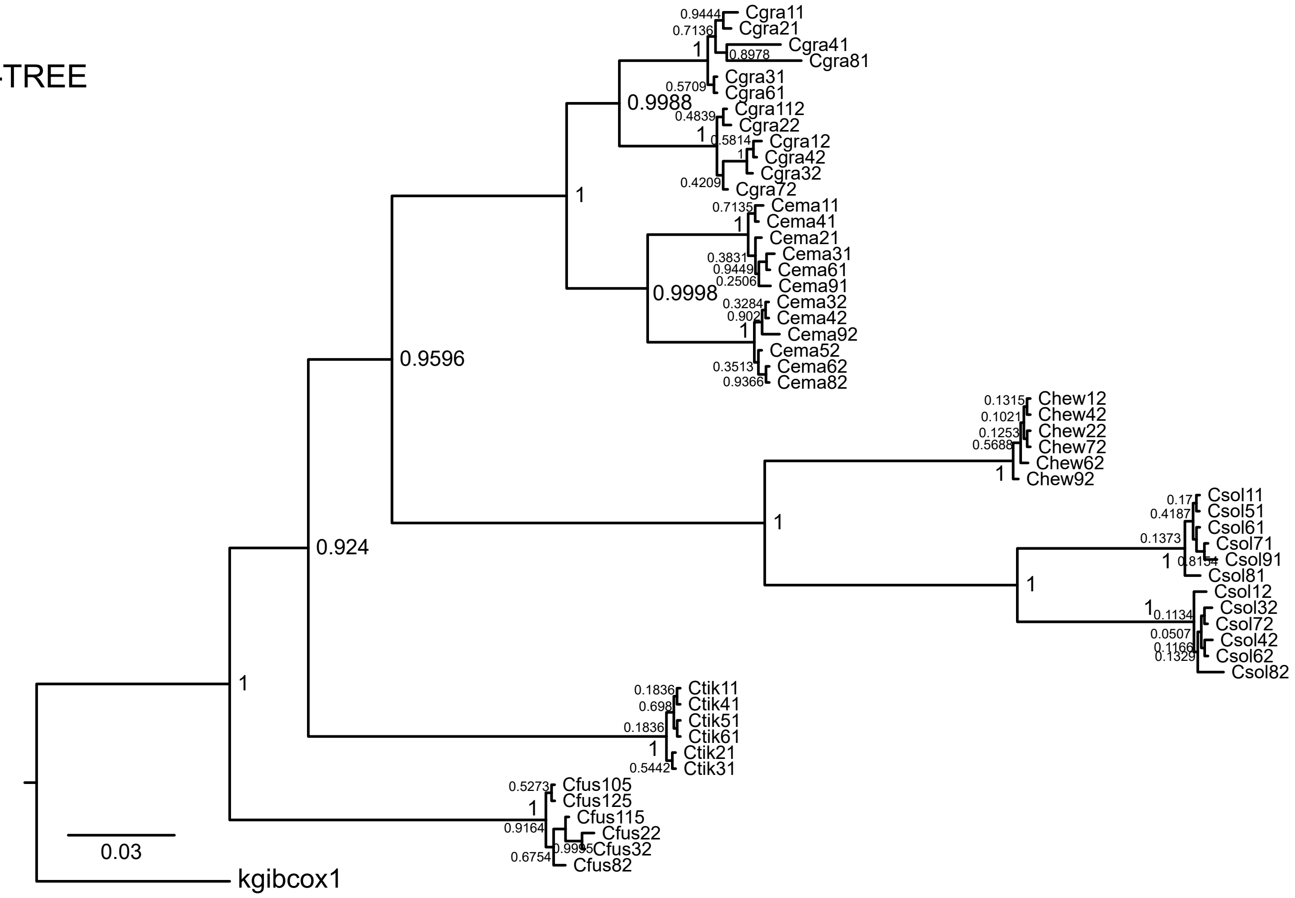

Supplementary Figure S1: Phylogenetic tree of fig wasps within genus *Ceratosolen* by ML (C) and Bayesian methods (D) based on CO1 sequences. Fig wasp species are abbreviated as follows: Cgra, *Ceratosolen gravelyi*; Cfus, *Ceratosolen fusciceps*; Ctik, *Ceratosolen* sp.; Chew, *Ceratosolen hewitti*; Cema, *Ceratosolen emarginatus*; Csol, *Ceratosolen solmsi*; Kgib, *Kradibia gibbosae*.
